# Retraction: *Drosophila yakuba – Tsc1*

**DOI:** 10.17912/micropub.biology.000470

**Published:** 2021-09-14

## Description

Lose, B; Myers, A; Fondse, S; Alberts, I; Stamm, J; Youngblom, JJ; Rele, CP; Reed, LK (2021). Private: *Drosophila yakuba – Tsc1*. microPublication Biology. 10.17912/micropub.biology.000407, was mistakenly published before peer-review was completed.

The published version of the article was removed from public view on www.microPublication.org as soon as the error was discovered and is now formally retracted.

The article was placed in the peer-review workflow and will be published as a new version should it pass peer- and editorial- review.

We regret this mistake.

